# Construction and validation of a risk assessment model for acute kidney injury in patients with acute pancreatitis in the intensive care unit

**DOI:** 10.1186/s12882-023-03369-x

**Published:** 2023-10-26

**Authors:** Ziming Jiang, Xiangyu An, Yueqian Li, Chen Xu, Haining Meng, Yan Qu

**Affiliations:** 1 Dalian Medical University, Dalian, 116000 Liaoning Province China; 2 Qingdao Hospital, University of Health and Rehabilitation Sciences (Qingdao Municipal Hospital), Qingdao, 266071 Shandong Province China; 3 Qingdao University, Qingdao, 266071 Shandong Province China; 4 Department of Critical Care Medicine, Qingdao Municipal Hospital, University of Health and Rehabilitation Sciences (Qingdao Municipal Hospital), Qingdao, 266071 Shandong Province China

**Keywords:** Acute Pancreatitis, Acute kidney injury, MIMIC-IV database, LASSO regression, Nomogram

## Abstract

**Background:**

To construct and validate a risk assessment model for acute kidney injury (AKI) in patients with acute pancreatitis (AP) in the intensive care unit (ICU).

**Methods:**

A total of 963 patients diagnosed with acute pancreatitis (AP) from the Medical Information Mart for Intensive Care IV (MIMIC-IV) database was included. These patients were randomly divided into training set (N = 674) and validation set (N = 289) at a ratio of 7:3. Clinical characteristics were utilized to establish a nomogram for the prediction of AKI during ICU stay. These variables were selected by the least absolute shrinkage and selection operation (LASSO) regression and included in multivariate logistic regression analysis. Variables with P-values less than 0.05 were included in the final model. A nomogram was constructed based on the final model. The predicted accuracy of the nomogram was assessed by calculating the receiver operating characteristic curve (ROC) and the area under the curve (AUC). Moreover, calibration curves and Hosmer-Lemeshow goodness-of-fit test (HL test) were performed to evaluate model performance. Decision curve analysis (DCA) evaluated the clinical net benefit of the model.

**Results:**

A multivariable model that included 6 variables: weight, SOFA score, white blood cell count, albumin, chronic heart failure, and sepsis. The C-index of the nomogram was 0.82, and the area under the receiver operating characteristic curve (AUC) of the training set and validation set were 0.82 (95% confidence interval:0.79–0.86) and 0.76 (95% confidence interval: 0.70–0.82), respectively. Calibration plots showed good consistency between predicted and observed outcomes in both the training and validation sets. DCA confirmed the clinical value of the model and its good impact on actual decision-making.

**Conclusion:**

We identified risk factors associated with the development of AKI in patients with AP. A risk prediction model for AKI in ICU patients with AP was constructed, and improving the treatment strategy of relevant factors in the model can reduce the risk of AKI in AP patients.

## Background

Acute pancreatitis (AP) is a form of acute abdominal distress that originates from the abnormal activation of pancreatic enzymes, resulting in digestive effects on the pancreas and surrounding organs. This condition is marked by local inflammatory reactions within the pancreas and can lead to organ dysfunction. AP is one of the most prevalent gastrointestinal diseases, causing significant physical, psychological, and economic burdens on patients [1]. Global incidence rates of acute pancreatitis range from 4.9 to 73.9 per 100,000 individuals, and the annual incidence rate is on the rise [2]. However, overall mortality rates have remained unchanged [1, 3, 4]. The diagnostic criteria for AP [5] include persistent upper abdominal pain, serum amylase and/or lipase concentrations at least three times higher than the upper limit of normal, and imaging results indicative of acute pancreatitis. Meeting two of these three criteria is sufficient for diagnosis. Most cases of AP are mild and self-limiting, requiring only brief hospitalization [6, 7]. Severe pancreatitis occurs in approximately 15–20% of patients [8] and is defined as the presence of organ failure lasting more than 48 h and/or death [1].

Acute kidney injury (AKI) is a prevalent complication observed in hospitalized patients, especially those who are critically ill. AKI is associated with high mortality and morbidity rates [9]. Moreover, AKI is considered a common and significant complication of acute pancreatitis, due to abdominal hypertension or abdominal compartment syndrome [10, 11], This condition is characterized by a sudden deterioration of renal function, reduced urine output, electrolyte and acid-base imbalances, fluid overload, and changes in the internal environment that negatively affect other organs [12].The occurrence of AKI in patients with acute pancreatitis may be linked to systemic inflammatory responses accompanied by increased vascular permeability, resulting in fluid exudation and accumulation in the peritoneal cavity and retroperitoneum, decreased blood supply to abdominal organs, and insufficient renal perfusion [13]. Patients with acute pancreatitis complicated by AKI have a higher mortality rate compared to those without AKI [14].Therefore, early recognition and risk assessment of AKI in patients with acute pancreatitis are crucial for improving disease prognosis.

Nomograms are graphical tools that utilize statistical prediction model to present and enhance the comprehension of clinical prediction models [15]. Despite their wide-ranging utilization, there are limited nomograms currently accessible for predicting the risk of AKI in patients suffering from acute pancreatitis. To address this research gap, the aim of our study is to develop a robust nomogram capable of accurately predicting the risk of AKI in critically ill patients with acute pancreatitis admitted to the ICU.

## Methods

### Data

The Medical Information Mart for Intensive Care(MIMIC) database is an expansive, open-access clinical repository that caters to critical care and emergency medicine domains. It is sourced from the intensive care system of Beth Israel Deaconess Medical Center(BIDMC) and archives hospitalization data concerning patients admitted to Massachusetts General Hospital between 2008 and 2019 [16, 17]. MIMIC-IV (version 2.0), which was released in June 2022, encompasses a range of improvements vis-à-vis its predecessor, MIMIC-III database, such as laboratory markers, medication records, recording of vital sign, SOFA score, SAPS II score, among others [18]. The authors have satisfactorily completed the requisite training for navigating the database and been conferred with the requisite certification (11,326,344).

### Study population

**Fig. 1:**
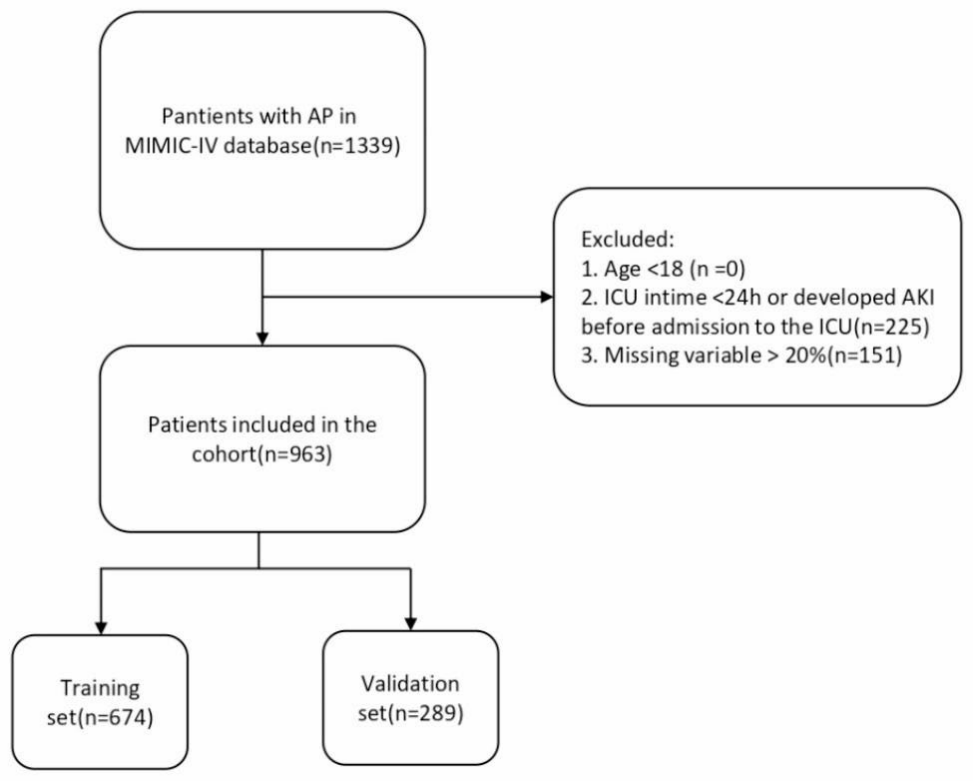
The flowchart of the study. Abbreviations: AP: Acute pancreatitis, ICU: Intensive care unit

Patients with AP were identified using the International Classification of Diseases, 9th Revision (ICD-9) code 5770 and the International Classification of Diseases, 10th Revision (ICD-10) code K85. Sepsis is defined as suspected or documented infection and an acute increase in SOFA score of ≥ 2 [19].Exclusion criteria encompassed patients who hadn’t been admitted to the ICU, stayed in ICU for less than 24 h, were below 18 years of age, had developed AKI before admission to the ICU, or had a clinical data missing rate in excess of 20%. The corresponding flowchart outlining the inclusion and exclusion criteria is depicted in Fig. 1.

### Definition and clinic variables

The primary outcome of our investigation was the incidence of AKI during ICU stay. To meet the criteria established by the Kidney Disease Improving Global Outcomes(KDIGO) classification system [20], AKI diagnosis required a rise in serum creatinine(Scr) by a minimum of 0.3 mg/dL within a period of 48 h, or an increase equal to or greater than 1.5 times from baseline within the previous seven days, the baseline creatinine was defined as the lowest creatinine level measured during the 7-day period prior to each AKI assessment, or urinary output less than 0.5 ml/kg/h for at least six consecutive hours. We collected detailed demographic information (Table 1), including age, gender, weight, as well as comorbidities such as hypertension, alcohol abuse, calculus gallbladder, pleural effusion, sepsis, myocardial infarction, chronic heart failure, chronic cerebrovascular disease, chronic lung disease, diabetes, chronic kidney disease, severe liver disease. We also documented scores pertaining to disease severity, including Sequential Organ Failure Assessment (SOFA), Glasgow Coma Scale (GCS), Systemic Inflammatory Response Syndrome (SIRS), and Charlson Comorbidity Index (CCI). Further, we analyzed clinical and laboratory indicators obtained within first 24 h after ICU admission. For variables measured multiple times, we included both the maximum and minimum values. This included measurements of heart rate, mean arterial pressure, respiratory rate, temperature, lactate, pH, partial pressure of oxygen, partial pressure of carbon dioxide, hemoglobin, mean corpuscular volume, platelet count, white blood cell count, mean corpuscular hemoglobin content, mean hemoglobin concentration, red blood cells, red cell distribution width, hematocrit, anion gap, bicarbonate, blood urea nitrogen, serum calcium, serum glucose, serum sodium, serum potassium, serum chloride, creatinine, albumin, international normalized ratio(INR), prothrombin time(PT), alanine aminotransferase(ALT), alkaline phosphatase(ALP), aspartate aminotransferase(AST), and total bilirubin. In addition, we considered urine output and fluid intake within 24 h, invasive mechanical ventilation within 24 h, use of vasopressors within 24 h, antibiotic use within 48 h, hospital length of stay, and ICU length of stay. Variables with a missing value greater than 20% were exclude from the analysis.

**Table 1:** Baseline characteristics of the patients with acute pancreatitis

Variable	level	Training cohort	Validation cohort	P value
		674	289	
Gender (%)	M	402 (59.6)	165 (57.1)	0.51
	F	272 (40.4)	124 (42.9)	
Age (years) (%)	< 65	434 (64.4)	170 (58.8)	0.12
	>=65	240 (35.6)	119 (41.2)	
Weight (kg)		83.33 [71.15, 100.84]	80.10 [70.00, 98.50]	0.09
Antibiotic (%)	no	192 (28.5)	88 (30.5)	0.60
	yes	482 (71.5)	201 (69.5)	
Hypertension (%)	no	558 (82.8)	249 (86.2)	0.23
	yes	116 (17.2)	40 (13.8)	
Alcohol abuse(%)	no	485 (72.0)	216 (74.7)	0.42
	yes	189 (28.0)	73 (25.3)	
Calculus gallbladder(%)	no	524 (77.7)	227 (78.6)	0.85
	yes	150 (22.3)	62 (21.4)	
PE (%)	no	625 (92.7)	268 (92.7)	1
	yes	49 (7.3)	21 (7.3)	
Sepsis (%)	no	233 (34.6)	109 (37.7)	0.39
	yes	441 (65.4)	180 (62.3)	
MI (%)	no	605 (89.8)	263 (91.0)	0.64
	yes	69 (10.2)	26 (9.0)	
CHF (%)	no	549 (81.5)	231 (79.9)	0.64
	yes	125 (18.5)	58 (20.1)	
CVD (%)	no	632 (93.8)	266 (92.0)	0.40
	yes	42 (6.2)	23 (8.0)	
CPD (%)	no	528 (78.3)	217 (75.1)	0.31
	yes	146 (21.7)	72 (25.9)	
Diabetes (%)	no	450 (66.8)	197 (68.2)	0.73
	yes	224 (33.2)	92 (31.8)	
Renal disease (%)	no	566 (84.0)	233 (80.6)	0.24
	yes	108 (16.0)	56 (19.4)	
Severe liver disease (%)	no	591 (87.7)	257 (89.9)	0.66
	yes	83 (12.3)	32 (11.1)	
CCI		4.00 [2.00, 6.00]	4.00 [2.00, 7.00]	0.73
SOFA		4.00 [2.00, 7.00]	4.00 [2.00, 6.00]	0.82
GCS		15.00 [14.00, 15.00]	15.00 [14.00, 15.000]	0.31
SIRS		3.00 [2.00, 4.00]	3.00 [3.00, 4.00]	0.45
HR max (beats/min)		111.00 [94.25, 127.00]	110.00 [93.00, 125.00]	0.44
HR min (beats/min)		90.00 [75.00, 106.00]	89.00 [76.00, 103.00]	0.50
MBP max (mmHg)		96.00 [86.00, 111.00]	96.00 [84.00, 109.00]	0.31
MBP min (mmHg)		68.00 [57.00, 79.000]	68.00 [57.00, 82.00]	0.53
RR max (breaths/min)		27.00 [23.00, 32.000]	27.00 [22.50, 32.00]	0.89
RR min (breaths/min)		16.00 [13.13, 19.00]	16.00 [13.50, 19.00]	0.33
TEM max (℃)		37.06 [36.72, 37.56]	37.06 [36.72, 37.56]	0.75
TEM min (℃)		36.72 [36.44, 37.11]	36.72 [36.39, 37.11]	0.95
Lactate (mmol/L)		1.70 [1.30, 2.80]	1.70 [1.20, 2.80]	0.50
PH		7.37 [7.29, 7.43]	7.37 [7.30, 7.42]	0.62
PO2 (mmHg)		82.00 [52.00, 127.00]	82.00 [50.00, 138.00]	0.91
PCO2 (mmHg)		39.00 [33.00, 46.00]	39.00 [33.00, 46.00]	0.87
Hb min (g/dL)		10.60 [8.90, 12.20]	10.30 [8.60, 12.20]	0.26
MCV max (fl.)		93.00 [88.00, 98.00]	94.00 [89.00, 99.00]	0.15
PLT min(×10^9^/L)		200.00 [133.25, 287.75]	185.00 [130.00, 282.00]	0.25
WBC max(×10^9^/L)		13.80 [9.70, 19.60]	12.900 [9.20, 19.10]	0.36
MCH max (pg)		30.45 [28.93, 32.10]	30.90 [29.20, 33.10]	0.02
MCHC max (g/L)		33.03 (1.68)	33.19 (1.87)	0.19
RBC max (×10^12^/L)		3.66 [3.13, 4.28]	3.55 [3.06, 4.15]	0.08
RDW max		15.00 [13.90, 16.58]	15.00 [13.80, 16.80]	0.60
HCT		34.30 [29.50, 39.48]	33.60 [29.50, 38.20]	0.32
Aniongap		15.00 [13.00, 18.46]	15.00 [12.50, 18.50]	0.14
Bicarbonate (mmol/L)		22.00 [18.00, 25.00]	22.00 [19.00, 25.00]	0.59
BUN max (mg/dL)		23.00 [13.00, 39.00]	21.00 [13.00, 35.00]	0.56
Ca max (mg/dL)		8.20 [7.60, 8.80]	8.10 [7.60, 8.80]	0.53
Glucose max (mg/dL)		140.00 [110.00, 195.50]	138.00 [109.00, 198.00]	0.83
Na max (mmol/L)		139.00 [136.00, 142.00]	138.00 [135.00, 142.00]	0.04
K max (mmol/L)		4.20 [3.80, 4.70]	4.30 [3.80, 4.70]	0.54
Cl max (mmol/L)		105.00 [100.00, 110.00]	104.00 [100.00, 109.00]	0.31
Cr max (mg/dL)		1.10 [0.70, 1.90]	1.00 [0.70, 1.70]	0.42
Alb (g/dL)		2.80 [2.30, 3.30]	2.80 [2.40, 3.20]	0.67
INR max		1.30 [1.20, 1.60]	1.30 [1.20, 1.60]	0.79
PT max (s)		14.50 [13.20, 17.40]	14.40 [13.10, 17.90]	0.61
ALT max (U/L)		48.00 [24.00, 117.75]	41.00 [22.00, 106.00]	0.30
ALP max (U/L)		110.00 [71.00, 198.00]	109.00 [69.00, 190.00]	0.85
AST max (U/L)		71.00 [34.00, 188.00]	58.00 [31.00, 125.00]	0.02
Tbil max (mg/dL)		1.00 [0.60, 2.70]	0.90 [0.50, 3.00]	0.84
Ventilation (%)	no	619 (91.8)	269 (93.1)	0.60
	yes	55 (8.2)	20 (6.9)	
Vasopressin (%)	no	476 (70.6)	211 (73.0)	0.50
	yes	198 (29.4)	78 (27.0)	
Fluid Intake (ml/kg/d) (%)	< 50	262 (38.9)	136 (47.1)	0.02
	>=50	412 (61.1)	153 (52.9)	
UO (ml/kg/h) (%)	< 0.5	216(32.0)	82 (28.4)	0.29
	>=0.5	458 (68.0)	207 (71.6)	
Los hospital (days)		16.79 [7.94, 30.14]	13.09 [7.50, 23.76]	0.01
Los ICU (days)		3.58 [1.94, 9.25]	3.21 [1.86, 7.00]	0.12
AKI (%)	no	183 (27.2)	85 (29.4)	0.52
	yes	491 (72.8)	204 (70.6)	

Abbreviations: AKI: Acute kidney injury, AP: Acute pancreatitis, PE: Pleural effusion, MI: Myocardial infarction, CHF: Chronic heart failure, CVD: Chronic cerebrovascular disease, CPD: Chronic pulmonary disease, CCI: Charlson comorbidity index, SOFA: Sequential organ failure assessment, GCS: Glasgow coma scale, SIRS: Systemic inflammatory response syndrome, HR: Heart rate, MBP: Mean blood pressure, RR: Respiratory rate, TEM: Temperature, HB: Hemoglobin, MCV: Mean corpuscular volume, PLT: Platelet count, WBC: White blood cell, MCH: Mean hemoglobin concentration, MCHC: Mean corpuscular hemoglobin content, RBC: Red blood cell, RDW: Red cell distribution width, HCT: Hematocrit, BUN: Blood urea nitrogen, ALB: Albumin, INR: International normalized ratio, PT: Prothrombin time, ALT: Alanine aminotransferase, ALP: Alkaline phosphatase, AST: Aspartate aminotransferase, TBIL: Total bilirubin, UO: Urine output, Los hospital: hospital length of stay, Los ICU: ICU length of stay

### Statistical analysis

The R statistical software (v4.2.3) was utilized to analyze the data in this study. To minimize information bias, variables with a missing rate of less than 20% were included. The MICE package [21] was employed to manage missing values of variables. Normally distributed continuous variables were presented as mean ± standard deviation (𝑥̄±𝑠), whilst non-normally distributed continuous variables were presented as median and quartiles [M(Q1, Q3)]. Categorical variables, on the other hand, were reported as percentages (%). The chi-square test or Fisher’s exact test was applied for analyzing categorical variables in intergroup comparisons, whereas continuous variables were assessed with paired t-test or Mann-Whitney U test, depending on the particular circumstance.

In the present investigation, the training set was employed to select important predictors for forecasting AKI risk using a suitable regression technique for high-dimensional data regression known as the least absolute and selection operator (LASSO) regression. The optimal penalty parameter λ value was determined by preforming a 10-fold cross-validation. Next, a multivariate logistic regression analysis was carried out based on the variables obtained from the LASSO regression, and variables with a P value of less than 0.05 were included in the model, resulting in six variables in the final model, namely weight, SOFA, white blood cell count, albumin, chronic heart failure, sepsis. To evaluate the performance of the model, the area under the receiver operating characteristic curve (AUC) was calculated for both the training and validation sets. Calibration curves and the Hosmer-Lemeshow goodness-of-fit test (HL test) were utilized to appraise the accuracy of the model. Furthermore, decision curve analysis was employed to assess the clinical utility of the prediction model.

## Results

### Patient characteristics

This study included a total of 963 patients, among whom 695 patients experienced AKI. Of these AKI patients, 599 were diagnosed based on urine output, 487 were diagnosed based on creatinine, and 391 were diagnosed based on both urine output and creatinine. In the training set, the cohort consisted of a total of 674 patients diagnosed with AP, of which more than half were also diagnosed with AKI(n = 491). The median weight of the patients was established as 83.33 kg, with a recorded history of CHF in 18.6% of the cases and 65.4% of the patients exhibit concomitant sepsis. The median SOFA score amounted to 4 points, while the median white blood cell count was measured at 13.8 × 10^9/L, and the median albumin level was 2.8 g/dL. Moreover, 32.05% of the subjects exhibited reduced urine output (defined as less than 0.5ml/kg/h), and 29.4% of the cases involved the use of vasopressors. Additionally, compared with patients without AKI, patients with AKI exhibited a prolonged length of hospital stay (19.10 days vs. 8.68 days) and ICU stay (4.70 days vs.2.03 days)(Table 2).

**Table 2:** Comparison of clinical outcomes in AKI patients and NO-AKI patients

level	No-AKI	AKI	P value
	268	695	
Los hospital (days)	8.68 [5.20, 15.50]	19.10 [9.83, 33.56]	< 0.01
Los ICU (days)	2.03 [1.48, 3.12]	4.70 [2.38, 11.87]	< 0.01

Abbreviations:AKI: Acute kidney injury, Los hospital: hospital length of stay, Los ICU: ICU length of stay

### Characteristics selection and development of a nomogram

**Fig. 2:**
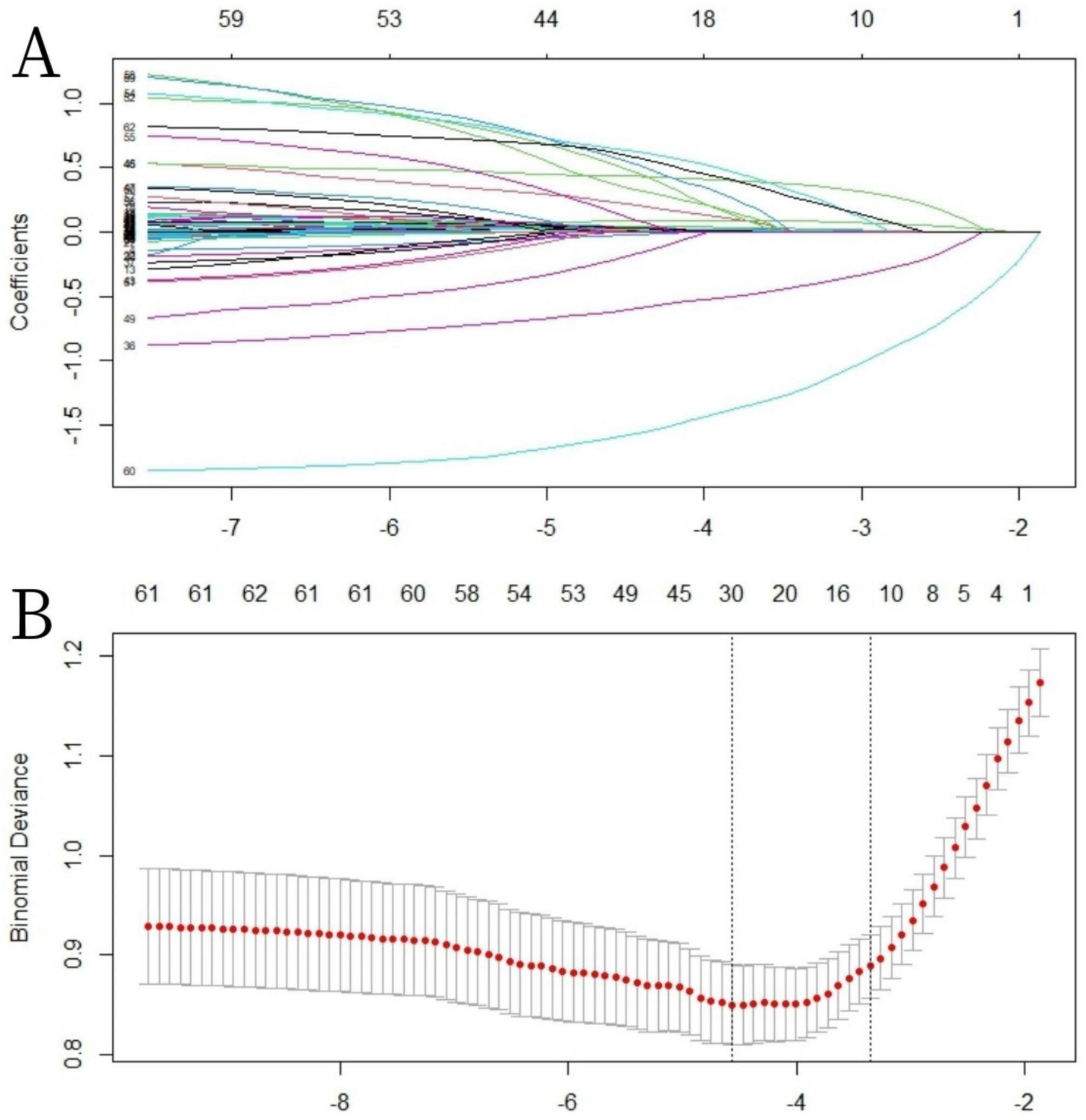
Demographic and clinical feature selection using the LASSO logistic regression model. **A** Tuning parameter (λ) selection using LASSO penalized logistic regression with 10-fold cross-validation. **B** LASSO coefficient profiles of the radiomic features. A coefficient profile plot was plotted versus the log (λ). Each colored line represents the coefficient of each feature

**Fig. 3:**
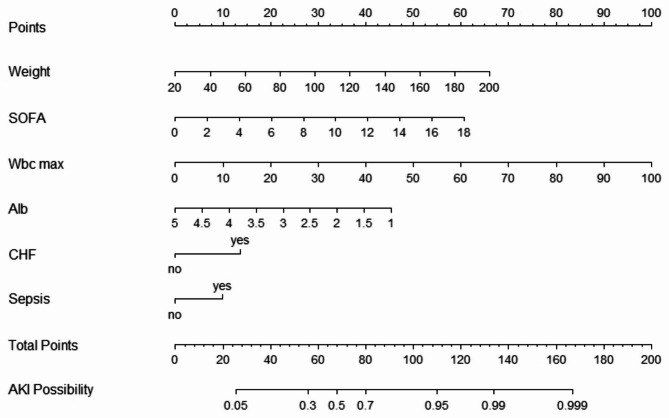
Nomogram to identify the risk of AKI following AP, based on logistic regression analysis. To assign scores to each variable, av vertical line is drawn upward to the “Point” axis, and the corresponding value is recorded. The sum of all predicted scores is then located on the “Total Point” axis. By drawing a line downward to the “AKI Possibility” axis, the likelihood of development AKI can be determined. The abbreviations used are as followings: SOFA: Sequential organ failure assessment, WBC: White blood cells, Alb: albumin, CHF: Chronic heart failure

A LASSO regression analysis was conducted on a total of 62 variables, ultimately selecting 11 variables for inclusion in the subsequent multivariable logistic regression analysis (Fig. 2). Of these 11 variables, six predictors were identified as significant contributors to the model(Table 3): weight (OR: 1.03; 95%CI 1.02–1.04), sepsis (OR: 1.89; 95%CI 1.23–2.92), chronic heart failure (OR: 2.53; 95%CI 1.35–4.73), SOFA (OR: 1.24; 95%CI 1.14–1.34), white blood cell count (OR: 1.07; 95%CI 1.04–1.1), albumin (OR: 0.48; 95%CI 0.35–0.66). Considering that urine output is one of the diagnostic criteria for AKI, we ultimately decided not to include it in the model. Based on these findings, a nomogram was constructed to predicted the probability of AKI incidence during the ICU stay of AP patients (Fig. 3).

**Table 3:** Multivariate logistic regression model of AKI in the training cohort

Variables	Multivariable analysis base on LASSO regression			Multivariable logistic model		
	β	OR (95%CI)	*p*	β	OR (95%CI)	*p*
Weight(kg)	0.02	1.02(1.01–1.03)	< 0.01	0.02	1.03(1.02–1.04)	< 0.01
Sepsis	0.50	1.66(1.04–2.63)	< 0.01	0.64	1.89(1.23–2.92)	< 0.01
CHF(yes)	0.84	2.33(1.19–4.58)	0.01	0.93	2.53(1.35–4.73)	< 0.01
SOFA	0.13	1.14(1.04–1.25)	< 0.01	0.21	1.24(1.14–1.34)	< 0.01
Wbc max(×10^9^/L)	0.06	1.07(1.03–1.1)	< 0.01	0.07	1.07(1.04–1.1)	< 0.01
Alb (g/dL)	-0.71	0.49(0.35–0.69)	< 0.01	-0.73	0.48(0.35–0.66)	< 0.01
Vasopressin (yes)	0.68	1.98(1.02–3.85)	0.04	0.49	1.64(0.88–3.06)	0.12
CCI	0.06	1.06(0.98–1.15)	0.16			
RR max (breaths/min)	0.04	1.04(1-1.08)	0.06			
RR min (breaths/min)	0.04	1.04(0.99–1.1)	0.13			
UO ( > = 0.5ml/kg/h)	-2.06	0.13(0.06–0.26)	< 0.01			

Abbreviations: AKI: Acute kidney injury, LASSO: the least absolute and selection operator, CHF: Chronic heart failure, SOFA: Sequential organ failure assessment, WBC: White blood cell, ALB: Albumin, UO: Urine output, CCI: Charlson comorbidity index, RR: Respiratory rate

### Apparent performance of the Nomogram

**Fig. 4:**
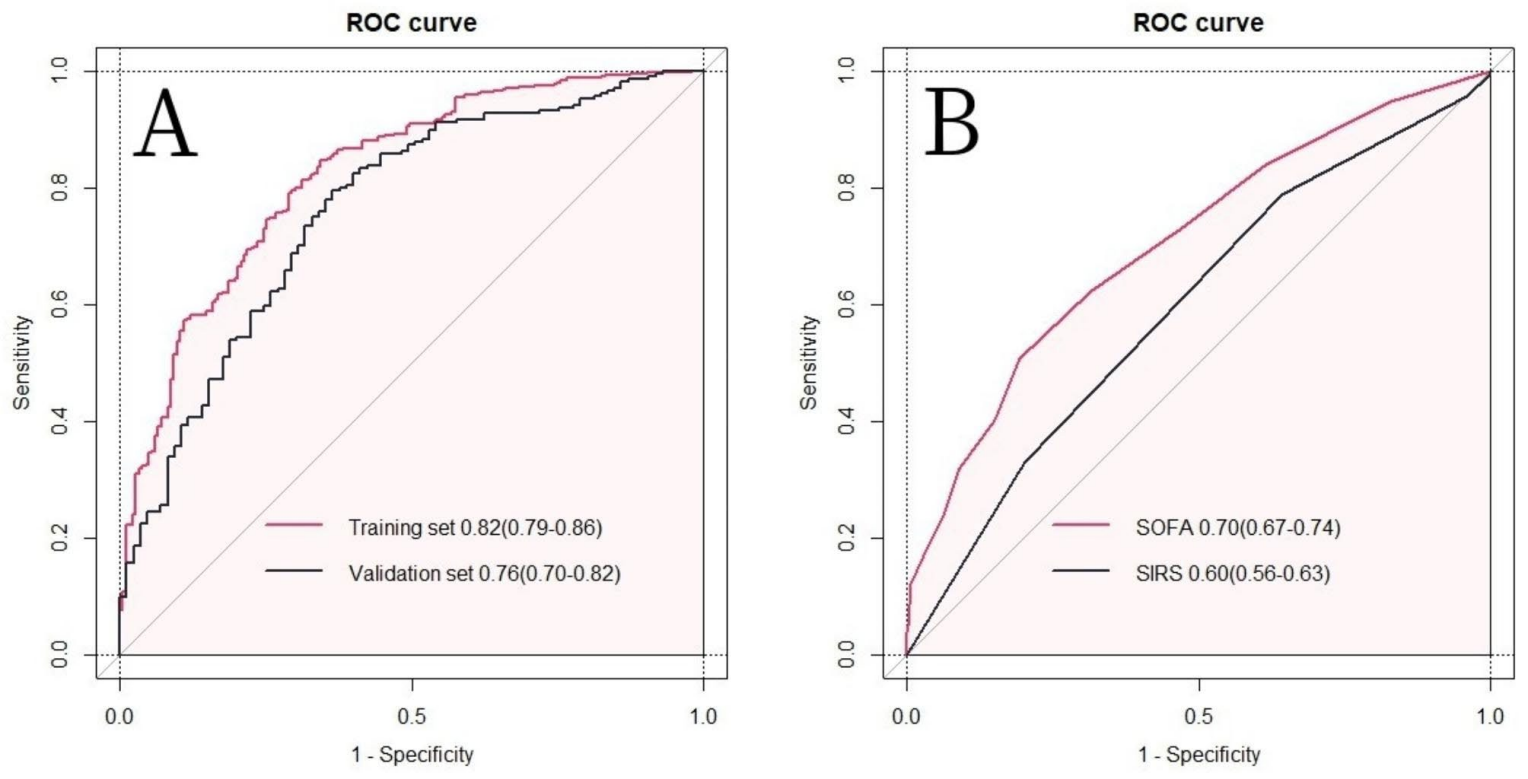
Receiver operating characteristic curve of the nomogram in the training set and validation set (**A**). Receiver operating characteristic curve of SOFA and SIRS (**B**) for predicting AKI in AP patients during the intensive care admission. Abbreviations: AUC: Area under the receiver operating curve, SOFA: Sequential organ failure assessment, SIRS: Systemic inflammatory response syndrome

**Fig. 5:**
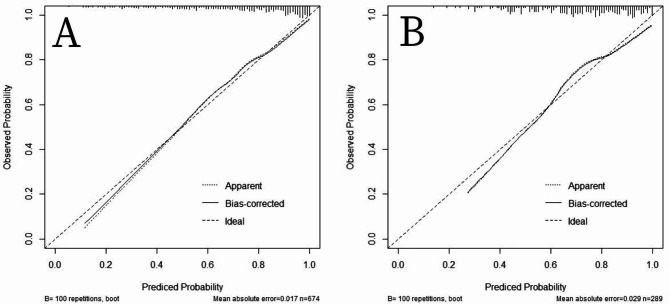
Calibration curves of the predicted nomogram in the training set (**A**) and validation set (**B**). The x-axis represents the predicted probability calculated by the nomogram, and the y-axis is the observed actual probability of AKI. The clino diagonal represents a perfect prediction by an ideal model. The solid curve represents the initial cohort and the dotted curve is bias corrected by bootstrapping (B = 100 repetitions), which demonstrates the performance of the predicted model

The AUC of the proposed model was evaluated in both the training and validation sets, yielding an AUC of 0.82 (95%CI:0.79–0.86) and 0.76(95%CI: 0.70–0.82), respectively (Fig. 4). Notably, the AUC values for both the training and validation sets were indicative of favorable diagnostic performance. Additionally, visual inspection of the calibration plot demonstrated satisfactory agreement between the predicted and observed AKI incidence rates, providing further support for the reliability of the model (Fig. 5).

### Clinical practice

**Fig. 6:**
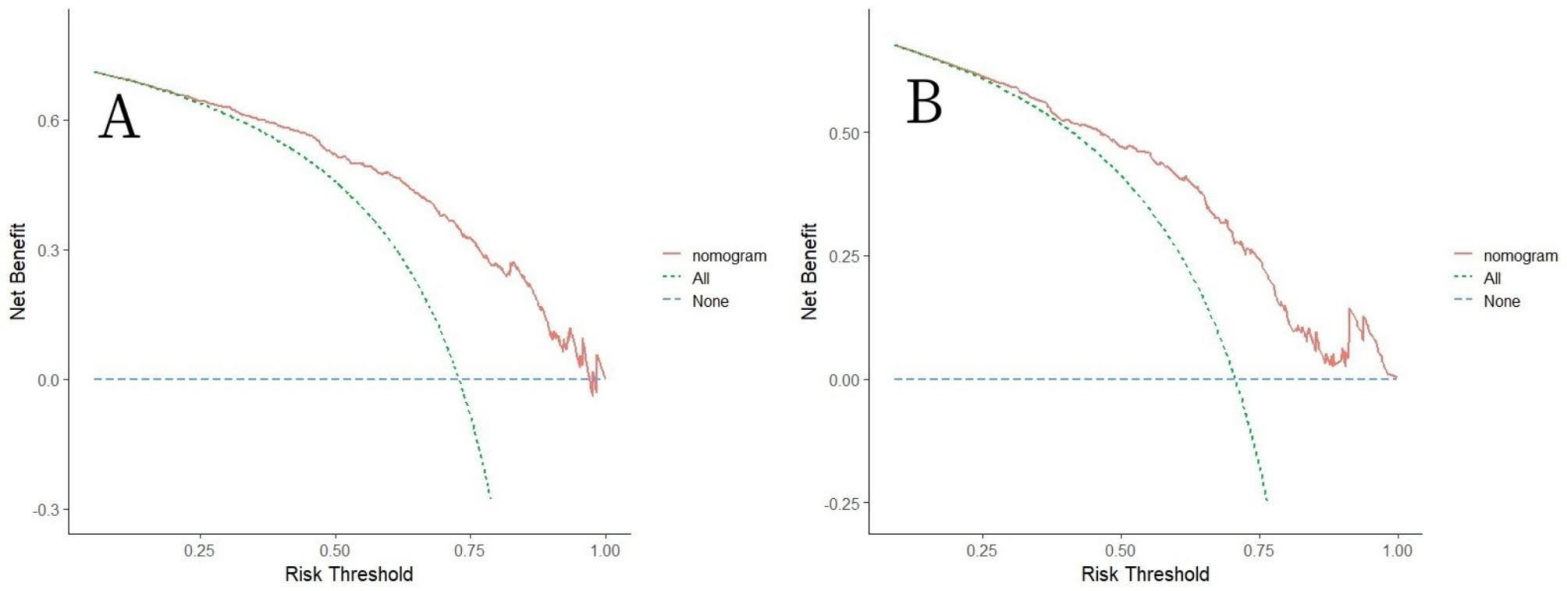
Decision curve analysis (DCA) of the nomogram in the training set (**A**) and the validation set (**B**). The horizontal line indicates no patients develop acute kidney injury (AKI), and the green oblique line indicates patients develop AKI. The red solid line represents the AKI risk nomogram. In DCA, the nomogram shows a more net benefit than full or no treatment across a threshold probability range. Abbreviations: DCA: decision curve analysis, AKI: acute kidney injury

Decision curve analysis (DCA) was conducted using both the training and validation datasets to evaluate the clinical usefulness of the proposed model (Fig. 6). The x-axis of the plot represents the proportion of patients not receiving intervention, which results in a net benefit of 0. Conversely, the diagonal line indicates that all patients receive intervention, resulting in a maximal net benefit. By setting a predicted probability threshold of 25–100% and 20–100% in the training and validation sets, respectively, the net benefit ranged from 0 to 62% and 0–62%, suggesting that the proposed model has the potential to provide clinical benefits by supporting individualized decision-making regarding AKI management.

## Discussion

This present study aimed to develop a nomogram for predicting the occurrence of AKI in the short term after AP by utilizing route information available in the ICU. Previous studies on AKI in AP patients have been limited by small sample sizes, and have not recorded data well [10].In a multicenter study conducted by Eric A J Hoste et al. [9], among 1802 ICU patients, 1032 patients developed AKI on the first day of ICU admission(57.3, 95%CI:55.0-59.6). Moreover, in another retrospective observational study, Devani et al. [14]reported an overall incidence of AKI of 7.9% among 3,466,493 hospitalized patients with acute pancreatitis. The main focus of this study was to investigate the development of AKI among patients in the ICU, specifically those diagnosed with AP. The findings revealed that a significant proportion (72.1%) of AP patients develop AKI in the ICU, which is notably higher than the incidence rate observed among hospitalized AP patients. Given the severity of the disease and the high incidence of AKI in the ICU, it was considered an acceptable finding. Furthermore, the study investigated the association between AKI and several outcome measures. It was observed that patients with AP complicated by AKI had longer hospital stays, and longer ICU stays compared to those who did not develop AKI(P < 0.01). These results suggest that the treatment efficacy for patients with AP who develop AKI is poorer and that the resulting economic burden is greater. To mitigate the negative outcomes associated with AP, it is necessary to develop a nomogram that can identify high-risk patients early on and understand the impact of relevant factors on outcomes. This study reinforces the importance of such an approach and highlights the need for further research in this area to improve treatment outcomes and reduce the economic burden associated with AP.

The nomogram development in this study incorporated six predictive factors, namely weight, sepsis, chronic heart failure, SOFA score, white blood cell count, albumin. The model achieved an AUC of 0.82 in the training set and 0.76 in the validation set, and calibration curve results indicated that the predicted values were consistent with the actual values. DCA revealed that utilizing the model to forecast AKI offered more benefits than either treating all patients.

Previous studies have linked obesity, a risk factor for AP [22], to increased likelihood of AKI among ICU patients [23]. This study confirmed such finding by suggesting that weight elevation is associated with higher chances of developing AKI in AP patients. Sepsis is defined as organ dysfunction resulting from the host’s deleterious response to infection. One of the most common organs affected is the kidneys [24]. The late-stage secondary infection of the pancreas and abdominal cavity is prone to sepsis following acute pancreatitis. Additionally, patients with chronic heart failure are predisposed to AKI due to inadequate renal functional reserve, alteration in hemodynamic status, low cardiac output or congestion, and drug effects, notably diuretics and rein-angiotensin system blockers [25]. The SOFA score, commonly used to evaluate organ dysfunction in critically ill patients, was identified as another predictive factor for AKI [26], alongside an increase in WBC count, which is a metric utilized in assessing the severity of AP [27]. The heightened risk of AKI seen with elevated WBC count could stem from the generation of reactive oxygen species by neutrophils, leading to the destruction of normal cells in inflamed tissues [28]. Hypoalbuminemia, a reflection of the severity of AP in hospitalized patients according to prior meta-analyses [29], was also found to be relevant with AKI in this case.

Several limitations of this study merit consideration. Firstly, the dataset was derived from a single center spanning the period from 2008 to 2019; thus, external validation of the model is necessary across diverse medical institutions to ascertain its generalizability. Secondly, sub-classification of AP was not conducted, and certain pertinent data such as triglycerides, lipase, amylase, and neutrophil-to-lymphocyte ratio were unavailable due to missing data. Finally given that this study relied on ICU patients with AP, its applicability in the regular ward setting may be limited. Inclusion of data from patients with pancreatitis in ordinary wards has the potential to enhance the model’s accuracy and overall utility.

## Conclusions

We identified risk factors associated with the development of AKI in patients with AP. This study developed a novel nomogram for predicting risk of AKI in ICU patients with AP, comprising weight, SOFA score, sepsis, chronic heart failure, white blood cell count, albumin. The proposed nomogram demonstrated favorable performance with respect to discrimination, calibration, and clinical applicability.

## Data Availability

The raw data is available from the first author and corresponding authors on reasonable request.
